# Predicting Unplanned Transfers to the Intensive Care Unit: A Machine Learning Approach Leveraging Diverse Clinical Elements

**DOI:** 10.2196/medinform.8680

**Published:** 2017-11-22

**Authors:** Ben Wellner, Joan Grand, Elizabeth Canzone, Matt Coarr, Patrick W Brady, Jeffrey Simmons, Eric Kirkendall, Nathan Dean, Monica Kleinman, Peter Sylvester

**Affiliations:** ^1^ The MITRE Corporation Bedford, MA United States; ^2^ Cincinnati Children's Hospital Cincinnati, OH United States; ^3^ Children's National Health System Washington, DC United States; ^4^ Boston Children's Hospital Boston, MA United States

**Keywords:** clinical deterioration, machine learning, data mining, electronic health record, patient acuity, vital signs, nursing assessment, clinical laboratory techniques

## Abstract

**Background:**

Early warning scores aid in the detection of pediatric clinical deteriorations but include limited data inputs, rarely include data trends over time, and have limited validation.

**Objective:**

Machine learning methods that make use of large numbers of predictor variables are now commonplace. This work examines how different types of predictor variables derived from the electronic health record affect the performance of predicting unplanned transfers to the intensive care unit (ICU) at three large children’s hospitals.

**Methods:**

We trained separate models with data from three different institutions from 2011 through 2013 and evaluated models with 2014 data. Cases consisted of patients who transferred from the floor to the ICU and met one or more of 5 different priori defined criteria for suspected unplanned transfers. Controls were patients who were never transferred to the ICU. Predictor variables for the models were derived from vitals, labs, acuity scores, and nursing assessments. Classification models consisted of L1 and L2 regularized logistic regression and neural network models. We evaluated model performance over prediction horizons ranging from 1 to 16 hours.

**Results:**

Across the three institutions, the c-statistic values for our best models were 0.892 (95% CI 0.875-0.904), 0.902 (95% CI 0.880-0.923), and 0.899 (95% CI 0.879-0.919) for the task of identifying unplanned ICU transfer 6 hours before its occurrence and achieved 0.871 (95% CI 0.855-0.888), 0.872 (95% CI 0.850-0.895), and 0.850 (95% CI 0.825-0.875) for a prediction horizon of 16 hours. For our first model at 80% sensitivity, this resulted in a specificity of 80.5% (95% CI 77.4-83.7) and a positive predictive value of 5.2% (95% CI 4.5-6.2).

**Conclusions:**

Feature-rich models with many predictor variables allow for patient deterioration to be predicted accurately, even up to 16 hours in advance.

## Introduction

Better prediction of clinical deterioration is a priority as many patients today get harmed when precursors go unrecognized, leading to potentially preventable morbidity, mortality, and cost. Over the last two decades, it has become increasingly clear that precursors to clinical deterioration commonly exist, and rapid response systems that detect and respond to early deterioration can improve outcomes [1-5].

Increased mortality and morbidity is associated with deterioration in patients who require an unplanned transfer from the nursing floor to the ICU (Intensive Care Unit). The mortality rate associated with unrecognized deterioration that results in a delay of ICU transfer and the need for resuscitation can be as high as 67% [6,7]. Missing precursors to deterioration reduces the window of opportunity and margin of error for effective intervention and increases the intensity and complexity of the required care.

Clinical EHR (electronic health record) systems and their rich, heterogeneous data provide opportunities for impactful secondary use [8,9]. Yet fully taking advantage of such large repositories of data is a challenge because of sheer complexity of the data [10]. Machine learning methods offer a promising set of techniques to address such challenges by providing statistically sound data-driven methods able to identify subtle patterns in data while remaining robust to problems in data quality and completeness [11].

Most machine learning methods for predicting deterioration have focused on logistic regression models preceded by careful variable selection [12,13]. Recently, more advanced machine learning approaches including nonlinear and nonparametric methods have been used [14]. These more powerful methods can accommodate larger feature sets and also identify implicit or explicit feature interactions. In many cases, however, model interpretability can suffer [15,16].

The purpose of this study was to develop highly accurate predictive models able to identify unplanned transfers to the ICU at least 6 hours before transfer. Critically, we leverage thousands of predictor variables, rather than dozens as is common in predicting adverse health events. We hypothesized that such complex models provide better accuracy at longer prediction horizons, providing more time and opportunity for clinicians to act to reverse deterioration.

## Methods

### Research Team

The MITRE Corporation together with three pediatric hospitals, Boston Children’s Hospital (BCH), Children’s National Health System (CNHS), and Cincinnati Children’s Hospital Medical Center (CCHMC) formed a partnership for the purpose of sharing data to uncover issues impacting patient safety. Each hospital contributed EHR data from 2011 to 2014 totaling >1 million patients and >8 million patient encounters, forming >7.2 TB of data across all three hospitals. Clinical data available from the three hospitals using 2 different EHR vendors was used to in our study to predict deterioration.

### Case Identification

Cases in our study involved instances of unplanned transfers from an inpatient ward to the ICU. The unit of analysis was the ICU transfer and not the patient, as each patient could experience more than one ICU transfer within the same hospital admission. The case identification proceeded in two phases. First, a set of *candidate cases* were identified from admission- discharge-transfer (ADT) data by selecting patient encounters that involved a stay on the nursing floor followed by a transfer to the ICU. Specifically, the candidate cases included ICU transfers originating from all nursing floors, excluding any transfers from the emergency department (ED), operating room (OR), postanesthesia care unit (PACU) or ICU and excluding any transfers to the neonatal intensive care unit (NICU).

**Figure 1:**
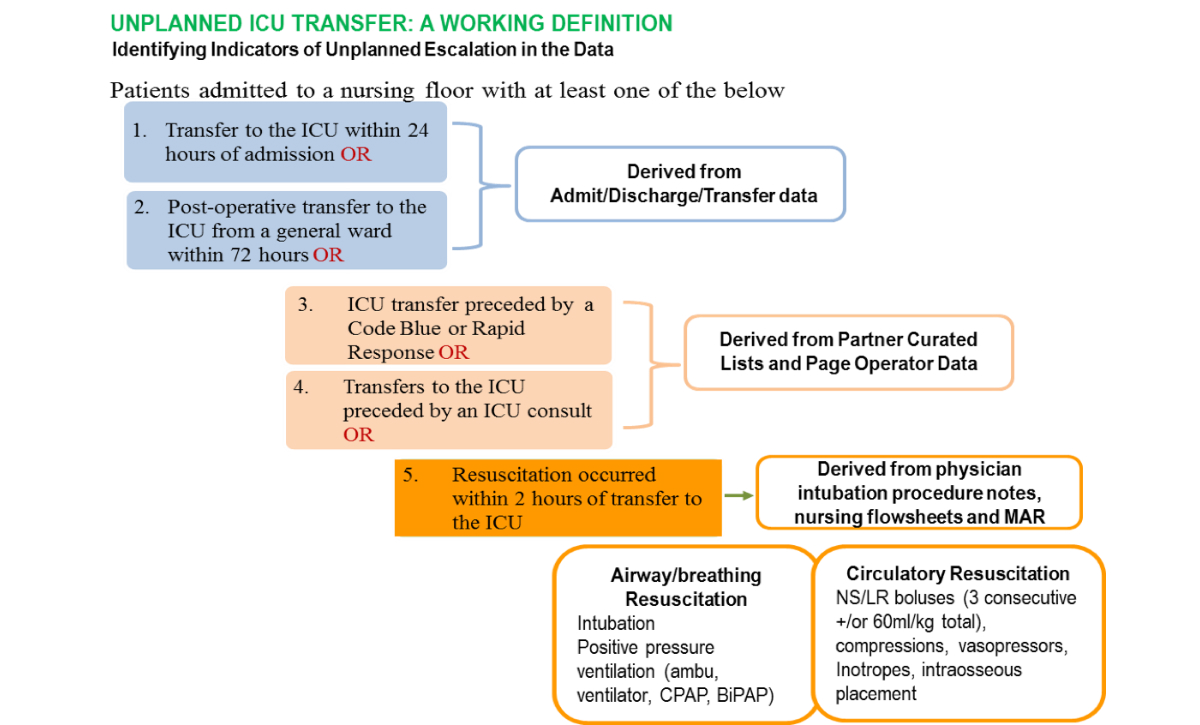
The five criteria involved in determining an “unplanned” intensive care unit (ICU) transfer. CPAP: continuous positive airway pressure; BiPAP: bilevel positive airway pressure; NS: normal saline; LR: lactated ringer; MAR: medication administration record.

From the candidate cases, we then developed a method for establishing whether a transfer was likely unplanned or not. Ideally, cases would be identified carefully by clinician review, as no variable or flag exists in the EHR to designate an unplanned transfer. To address this challenge, our team, which included clinicians at three hospitals, identified a set of five criteria to establish our case cohorts through objective, heuristic means. Unplanned transfers were identified as transfers to the ICU meeting one or more of the criteria (see Figure 1). This working definition of unplanned ICU transfer is the result of prior work in the literature [17] combined with knowledge gained from each institution’s experience.

We further subdivided the list of cases into those patients who experienced a critical deterioration event (CDE) along with an unplanned transfer to the ICU. CDE was defined as an unplanned floor to ICU transfer with invasive or noninvasive positive pressure ventilation, vasopressors, fluid resuscitation, or other emergent procedures 2 hours before and 12 hours post transfer [5]. The prediction model was aimed at predicting unplanned transfers; however, the CDE subgroup was important in understanding the connection between unplanned transfers and critical deteriorations.

### Identification of the Control Group

Controls were sampled from the set of patient visits where the patient spent at least 24 hours on an inpatient floor and was never transferred to the ICU. Sampling was done by ensuring that the ratios of ages and diagnoses were similar between the case and control population. Diagnoses were determined by discharge diagnosis according to ICD-9 (International Classification of Diseases-9). This sampling scheme was designed to balance the need for controls to be representative of the inpatient floor population, yet also to ensure that the control population did not differ from the case population in systematic ways. We removed patients from cases and controls that spent less than 8 hours on the floor. Table 1 provides the counts for the cases and controls across the three institutions.

**Table 1:** Counts for cases and controls across three institutions.

Dataset		BCH^a^	CCHMC^b^	CNHS^c^
**Training**				
	Cases	1163	1090	546
	Controls	6448	6170	3893
**Evaluation**				
	Cases	326	478	324
	Controls	1878	1353	1339

^a^Boston Children’s Hosptial.

^b^Cincinnati Children’s Hospital and Medical Center.

^c^Children’s National Health System.

### Clinical Element and Feature Extraction

Data preparation involved two primary stages before creating data instances for training and evaluating predictive models. The first stage involved pulling clinical element data out of underlying vendor database tables with a complex schema into a simplified set of database tables through a set of Structured Query Language (SQL) queries. The clinical element categories included vitals, laboratory results, acuity scores (eg, existing early warning score or nurse acuity calculations) and nursing assessments. An overview of the clinical elements used in our study, specific to data from Cincinnati Children’s are summarized in Table 2. Clinical elements based on patients’ vitals were standardized across the three hospitals. The other types of clinical elements, especially acuity and nursing assessments differed across the institutions because of different EHR systems and/or different customizations made by each institution. No attempt was made to standardize such elements. Although laboratory results would have been possible to harmonize across the institutions, acuity scores did not map from one institution to another. Nursing assessments provided even more variability; besides a lack of a one-to-one mapping between institutions, nursing assessments sometimes used values chosen from a fixed set (in a drop-down menu) and in other cases allowed for free text.

**Table 2:** Summary of clinical elements.

Clinical category	Clinical elements
Vitals	Temperature
	Heart rate
	Respiratory rate
	Systolic blood pressure
	Oxygen saturation
Laboratory results	Sodium
	Potassium
	Glucose
	Creatinine
	Bicarbonate
	White blood cell count
	Hermatocrit
	Hemoglobin
Acuity scores	PEWS^a^ total score
	Total acuity score
	Acuity level
Nursing assessments	Braden risk
	Activity
	Adult Glasgow coma score
	Audible sounds w/o stethoscope
	Best verbal response
	Brachial bilateral pulse
	Brachial left pulse
	Brachial right pulse
	Cardiac
	Cardiovascular
	Central perfusion cap refill
	Cough
	Eye opening
	Faces pain classification
	Faces pain score
	Femoral bilateral pulse
	Femoral left pulse
	Femoral right pulse
	FLAAC^b^ activity
	FLAAC consolability
	FLAAC cry/face/legs
	FLAAC pain classification
	FLAAC total pain score
	Fluid balance
	Friction sheer
	Heart rate/rhythm
	Patient experiencing pain?
	Level of consciousness
	Left lower extremity perfusion cap
	Left upper extremity perfusion cap refill
	Minimum stimulus to invoke response
	Mobility
	Moisture
	Neurological
	Neurovascular check
	NRS^c^ pain classification
	Nutrition
	Orientation level
	Orientation
	Ped Glasgow coma score
	Perfusion cap refill
	Perfusion color
	Perfusion skin temperature
	Peripheral pulses
	PERRLA^d^
	Pupil reaction
	Respirations/respiratory
	Respiratory status
	Response to stimuli
	Retractions
	Rhythm
	Right lower extremity perfusion cap
	Right upper extremity perfusion cap refill
	Secretion/sputum color
	Skin within normal limits
	Temperature condition
	Total pain score for site
	Upper perfusion cap refill
	Work of breathing

^a^Pediatric Early Warning Score.

^b^Faces, Legs, Activity, Cry, Consolability.

^c^Numeric Rating Scale.

^d^Pupils, Equal, Round, React to Light, Accomodation.

**Table 3:** Feature types used to construct features from clinical elements.

Feature type description				Feature examples
**Vitals**				
	Linear regression slope over scalar vitals of given type			HR^a^ slope=−2.1
	Magnitude of linear regression slope			HR magnitude slope=2.1
	Sign of slope of linear regression			HR slope is negative
	Binned category C1-C4 of MAXIMUM value			Maximum HR is C4
	Binned category C1-C4 of MINIMUM value			Minimum HR is C1
	Binned category C1-C4 of AVERAGE value			Average HR is C3
	Binned category C1-C4 of NEWEST (most recent) value			Newest HR is C4
	Binned category C1-C4 of OLDEST (least recent) value			Oldest HR is C1
	Normalized histogram values over categories computed by counting category assignments for each measurement and normalizing to 1			HR C1 Histogram=0.3; HR C2 Histogram=0.4; HR C4 Histogram=0.3
**Labs**				
	Category (Low, Normal, High) pairs: 2nd Newest and Newest			Glucose low≥normal
	Change or lack of change in category (Low, Normal, High) from 2nd Newest to Newest			Creatinine high≥high
	**Binned percentage change in value from Oldest to Newest value**			WBC^b^ new/old>1.5
		Newest/Oldest>{1.25, 1.5, 2.0, 3,0}		
		Newest/Oldest<{0.8, 0.67, 0.5, 0.33}		
	**Binned percentage change in value from 2nd Newest to Newest value**			Glucose new/2nd<0.5
		Newest/Oldest>{1.25, 1.5, 2.0, 3,0}		
		Newest/Oldest<{0.8, 0.67, 0.5, 0.33}		
	Attribute type is present in prediction window 1 or more times			Glucose is present; WBC is present
	Last category (Low, Normal, High)			Last WBC is high
**Acuity**				
	Score MINIMUM is 0 or value>{0, 1, 2, 3, 4, 5, 6, 7, 8, 9}			Minimum PEWS^c^ score is 0
	Score MAXIMUM is 0 or value>{0, 1, 2, 3, 4, 5, 6, 7, 8, 9}			Maximum PEWS score>0
	NEWEST score is 0 or value>{0, 1, 2, 3, 4, 5, 6, 7, 8, 9}			Newest PEWS score>0
	Linear regression slope over scores			Slope PEWS score=-1.5
	Linear regression slope over scores over last 6 hours			Slope PEWS score last 6 hours=3.1
	Magnitude of slope over scores			Magnitude slope PEWS score=1.5
	Magnitude of slope over scores over last 6 hours			Magnitude slope PEWS score=1.5
	Number of measurements of type over last 6 hours>{0, 1,2,4,6,10} (multiple overlapping features included)			Number of PEWS Measurements >0; Number of PEWS Measurements >1; Number of PEWS Measurements >2
**Assessments**				
	Nursing assessment attribute value pair (whether value is scalar or a string)			Cough is productive; Mobility is 1; Mobility is 2
	NEWEST assessment attribute-value pair for given attribute			Newest mobility is 2

^a^Heart rate.

^b^White blood cell count.

^c^Pediatric early warning score.

Clinical elements present within the prediction window for each clinical element type were used for feature extraction. The prediction window for vitals included the time frame of 24 hours leading up to the prediction time and for all other elements the length of the prediction window was 72 hours. From raw clinical elements, extracted features aim to capture the state of the patient and patient trajectory. Many of our features using vitals follow the approach taken by Zhai et al [12]. For example, vitals were binned into risk categories C1 to C4. Features are then derived from these categorized/binned vitals. The types of features derived from the various clinical elements are summarized in Table 3 **.** Noteworthy is that we made no attempt to impute missing values.

### Machine Learning Methods

Our experiments used logistic regression models and a nonlinear extension to logistic regression in the form of multilayer perceptrons (MLPs), also known as feed-forward neural networks. Neural networks have seen a resurgence in recent years with improved techniques to train them efficiently and effectively.

For binary classification, logistic regression can be written as:

p(y=1|x) = logistic(x) = 1/ (1 + exp(−wx)) (1)

where *w* is the set of weights (or coefficients) in the model and x represents a vector of input variables, that is, features. Hidden layers consist of sets of neurons; each layer can be viewed as successive (nonlinear) transformations of the input, each having the form:

H_i_(z) = g(W_i_(z)) (2)

Where *z* is the input vector to layer *i*, *g* is an activation function and *W*_i_ is a matrix of weights. In our models here, we use a rectified linear activation function of the form *g* (*x*)=max(0, *x*). Given this form, a MLP with *n* hidden layers can be written as:

*p* (*y*=1| *x*) = *logistic* (*H*_n_(*H*_n-1_(…(*H*_1_(*x*))))) (3)

As with logistic regression, the model is fit by maximizing the likelihood of the training data . However, given the large number of parameters in our models caused by so many features, there is a strong tendency to *overfit* the training data leading to poor generalization on unseen data. Accordingly, we heavily regularize our models using L1 and L2 regularization terms [18], their joint use sometimes referred to as elastic net regularization. L1 regularization is especially useful as it implicitly performs feature selection. This is beneficial in our case with potentially many irrelevant features [19]. MLPs are even more prone to overfitting as they include more parameters and capture complex nonlinear interactions between the inputs. Our experiments using MLPs make use of dropout [20], a technique in which a certain percentage of the neurons are randomly elided upon processing each data point during training.

Regularization can be achieved by adding penalty terms to the likelihood based on the L1 and L2 norms of the model weights. The penalized log-likelihood has the form:

L(*D*, *W*) =∑_i=1_[log *p* (*y*= *y*^(i)^| *x*^(i)^)] − *a*_1_|| *W* ||_1_ − *a*_2_|| *W* ||_2_ (4)

where *W* refers to *all* the weights in the model (including any hidden layer weights) and where *a*_1_ and *a*_2_ are “hyper”-parameters that determine the “strength” of the two regularizer components: || *W* ||_1_ denoting the L1 norm of the parameters and || *W* ||_2_ the L2 norm. These regularizers penalize large-magnitude weights and prevent the model from fitting the training data too closely at the expense of its ability to generalize. Modern machine learning techniques rely heavily on regularization to develop accurate prediction models with large numbers of features and modest amounts of training data.

Estimating the parameters for all models (logistic regression and MLPs) was done by maximizing the penalized likelihood with stochastic gradient descent [21,22]. All machine learning models were trained and used for prediction with the Mandolin machine learning toolkit available as open source on Github.

### Model Preparation

The training data used to construct our models leveraged patient encounters from January 1, 2011 through December 31, 2013. Separate models were trained for each institution because clinical elements are not standardized across EHR systems. Model settings such as the regularization coefficients, the number of hidden layers for MLP models, and the number of training iterations were tuned using 5-fold cross validation on the training set. Given the low prevalence of unplanned transfers, we subsampled the controls so that our training data had roughly a 1:5 ratio of cases to controls. We measured the area under the receiver operating characteristic (ROC) curve, the specificity at the threshold corresponding to 80% sensitivity, and also computed the estimated positive predictive value (PPV) given the overall 1.3% prevalence in our dataset. The estimated PPV was derived from the sensitivity, specificity, and prevalence [23].

### Experimental Design

We carried out three sets of experiments across all three institutions to measure the contributions of four different clinical element types. The first set of experiments looked at the performance of predictive models using only clinical elements of a single type. A second set of experiments looked at performance when features from each the clinical element types were added successively, in the order: *vitals, lab results, acuity scores,* and *nursing assessments*. Finally, we carried out a set of ablation experiments comparing the full model, making use of all features with feature sets constructed by removing features for each clinical element type separately. These experiments were carried out with regularized logistic regression.

A key concern in the practical use of a predictive model for detecting patient deterioration is how sensitive the model might be to varying lengths of time between when a prediction is made and when a patient is transferred to the ICU or prediction horizons. For controls, the prediction horizon is the time between when the prediction is made and the patient leaves the floor.

We provided results on experiments training the model to predict deterioration at prediction horizons varying from 1 hour to 16 hours, at 1-hour intervals. Evaluation on the test set was done using the same prediction horizon as was used to train the model.

We examined how well models with different feature sets performed across different prediction horizons.

This set of experiments examined how well models trained to identify deterioration with a given prediction horizon performed when evaluated across different prediction horizons. For example, how a model fared when asked to predict deterioration 16 hours in advance if it was trained to identify deterioration with just a 2-hour prediction horizon. Conversely, how might a model predict risk of deterioration for a patient just 2 hours away from an unplanned transfer if trained to identify deterioration 16 hours in advance.

Finally, experiments were carried out to measure the effect of regularization on logistic regression models, reducing the number of input features by feature selection and also provide more detailed comparison of MLP models versus logistic regression.

All models were binary classifiers designed to predict whether a patient will have an unplanned transfer to the ICU or not. Evaluation is carried out on the test data from 2014. Our primary evaluation metric is the area under the ROC curve. We also considered the models’ specificity at 80% sensitivity and examined the PPV at this cut-point, assuming a prevalence of 1.3% which matched the prevalence of deterioration across the three institutions.

### Ethics Approval

The study was reviewed and approved by the institutional review boards at Boston Children’s Hospital, Children’s National Medical Center, and Cincinnati Children’s Hospital Medical Center.

## Results

### Clinical Element Analysis

Our experiments followed the case and control selection methodologies described, and subsampled the controls. The total case and control counts are shown in Table 1. Primary results are detailed in Table 4. The feature configurations prefixed with All- *X* involved using all features except for those of type *X*. Interestingly, removing any single feature type from all available features generally resulted in minor, nonstatistically significant, reductions in the area under the ROC curve. This held except for the case of removing nursing assessments, which resulted in statistically significant degradations for CCHMC and CNHC but not BCH.

The prediction horizon used for training models and evaluating them was 6 hours. The models used all available features for all experiments and all models were regularized logistic regression except for the rows with MLP denoting a multilayer perceptron model. All logistic regression models across all features sets and institutions used a_1_=0.001 and a_2_=0.01 (see the likelihood equation above); these values were determined empirically using 5-fold cross validation on the training data. The MLP experiments here used three hidden layers with the rectified linear activation function. The first, second, and third layers had 60, 40 and 40 nodes, respectively. Each layer used a 50% dropout rate [20], with L1 regularization (a_1_=0.0003). Again, these model settings were determined through 5-fold cross validation experiments on the training sets. As with regularized logistic regression, the same MLP model settings were used across all three institutions’ datasets.

### Varying the Prediction Horizon

**Figure 2:**
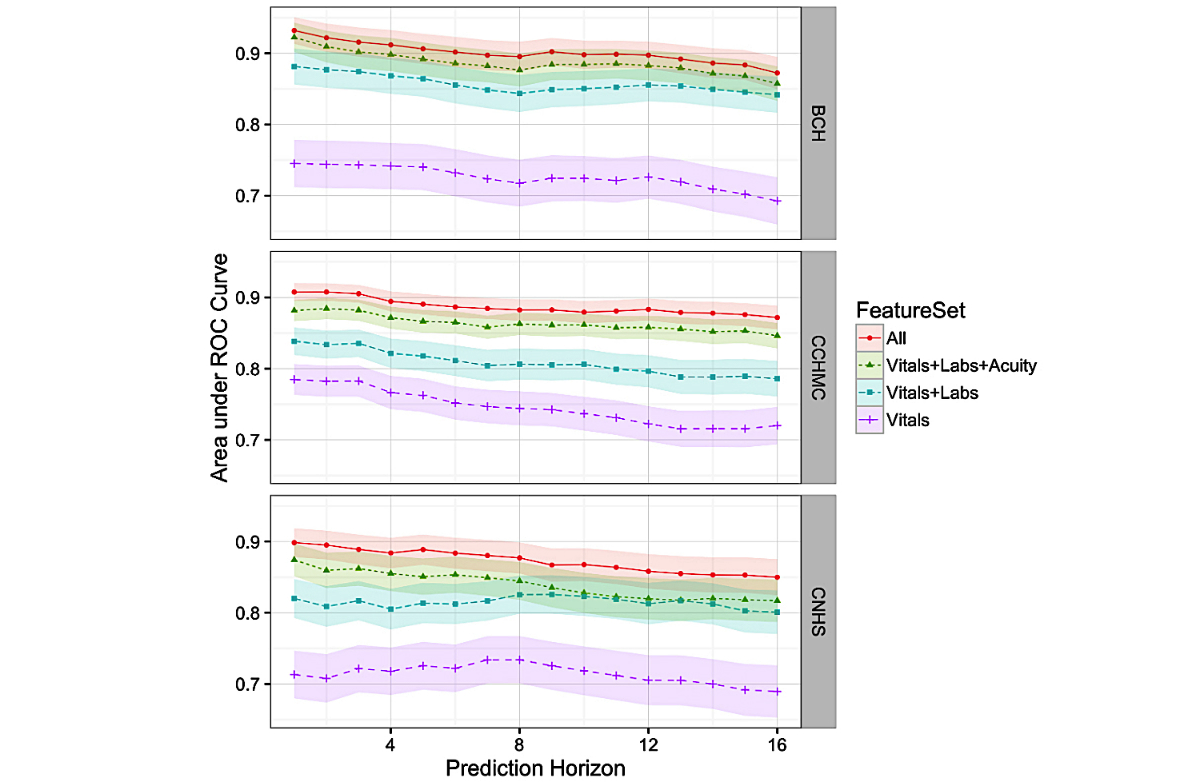
Model performance with increasingly complex (additive) feature sets across prediction horizons, including 95% CIs. ROC: receiver operating characteristic; BCH: Boston Children’s Hospital; CCHMC: Cincinnati Children’s Hospital and Medical Center; CNHS: Children’s National Health System.

**Figure 3:**
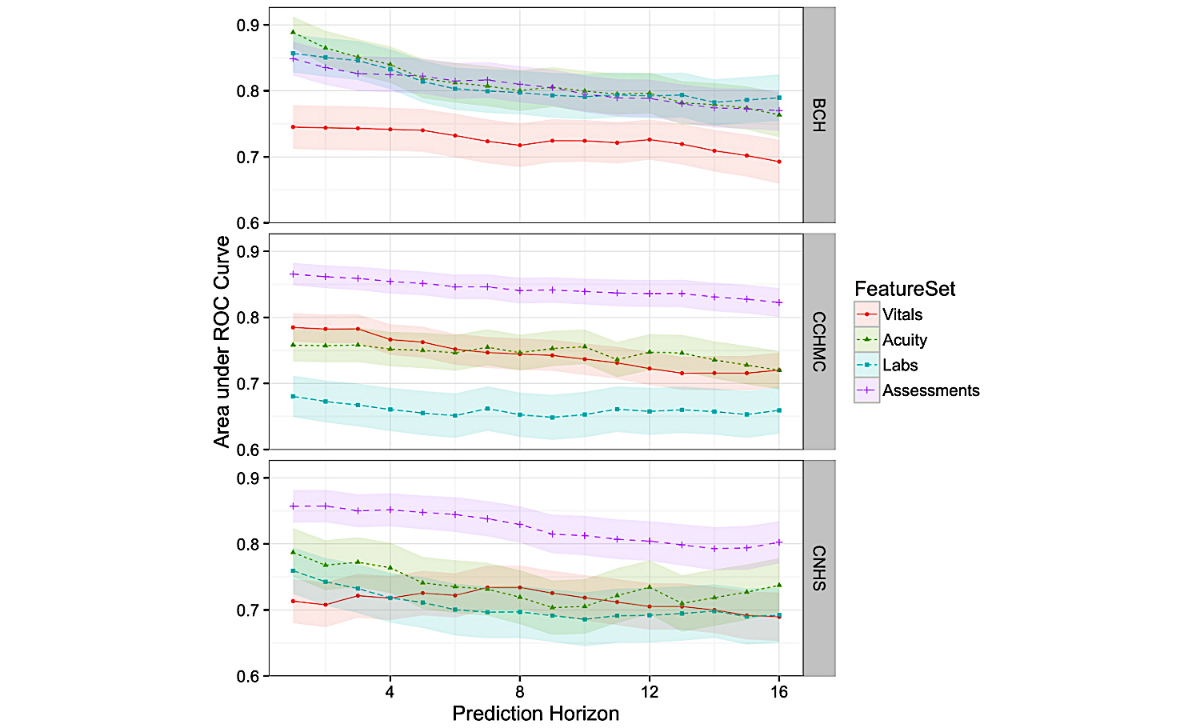
Performance of models with individual feature sets across prediction horizons, including 95% CIs. ROC: receiver operating characteristic; BCH: Boston Children’s Hospital; CCHMC: Cincinnati Children’s Hospital and Medical Center; CNHS: Children’s National Health System.

Although our focus involved predicting deterioration 6 hours before the event, we also considered how well the models performed across different prediction horizons. Additionally, we wanted to further examine the contributions of different groups of features from the various clinical elements to determine how particular feature groups performed at each horizon interval. Figure 2 shows the results for models trained and evaluated at prediction horizons ranging from 1 to 16 hours. We examined four different models where feature groups were successively added, starting with *vitals*, then adding *labs*, then *acuity* and finally *assessments* to arrive at the full model. As we were also interested in understanding how each feature group performed independently of the others; Figure 3 presents results over different prediction horizons considering at each group of features separately. These results show robustness in the models’ ability to predict deterioration even 10 to 16 hours before the event.

**Figure 4:**
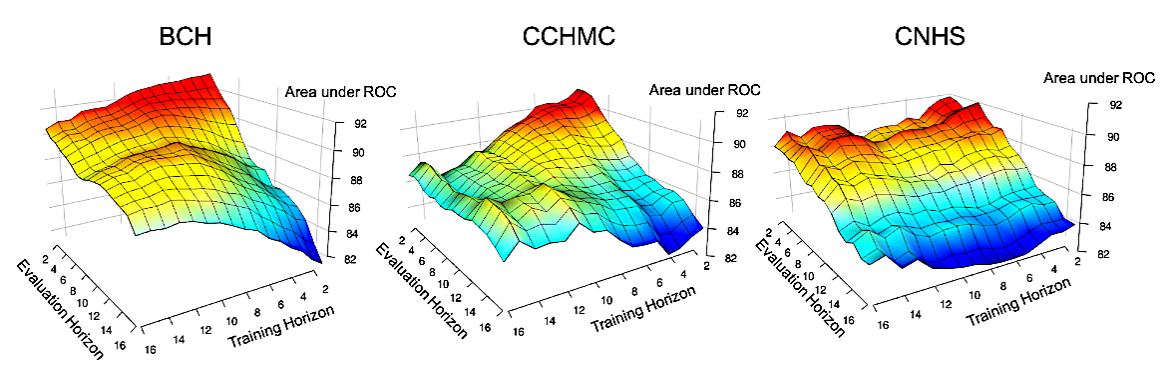
Area under receiver operating characteristic (ROC) curve when training and evaluating models across prediction horizons ranging from 1 hour to 16 hours. BCH: Boston Children’s Hospital; CCHMC: Cincinnati Children’s Hospital and Medical Center; CNHS: Children’s National Health System.

In addition, we examined how well a model trained for a particular prediction horizon performed when evaluated against varying prediction horizons. We carried this out by looking at a set of cross horizon experiments taking the 16 models trained across prediction horizons from 1 to 16 hours (using all available features) and evaluating each of those models against horizons ranging from 1 to 16. These results are presented as surface plots shown in Figure 4.

### Model Comparison

**Figure 5:**
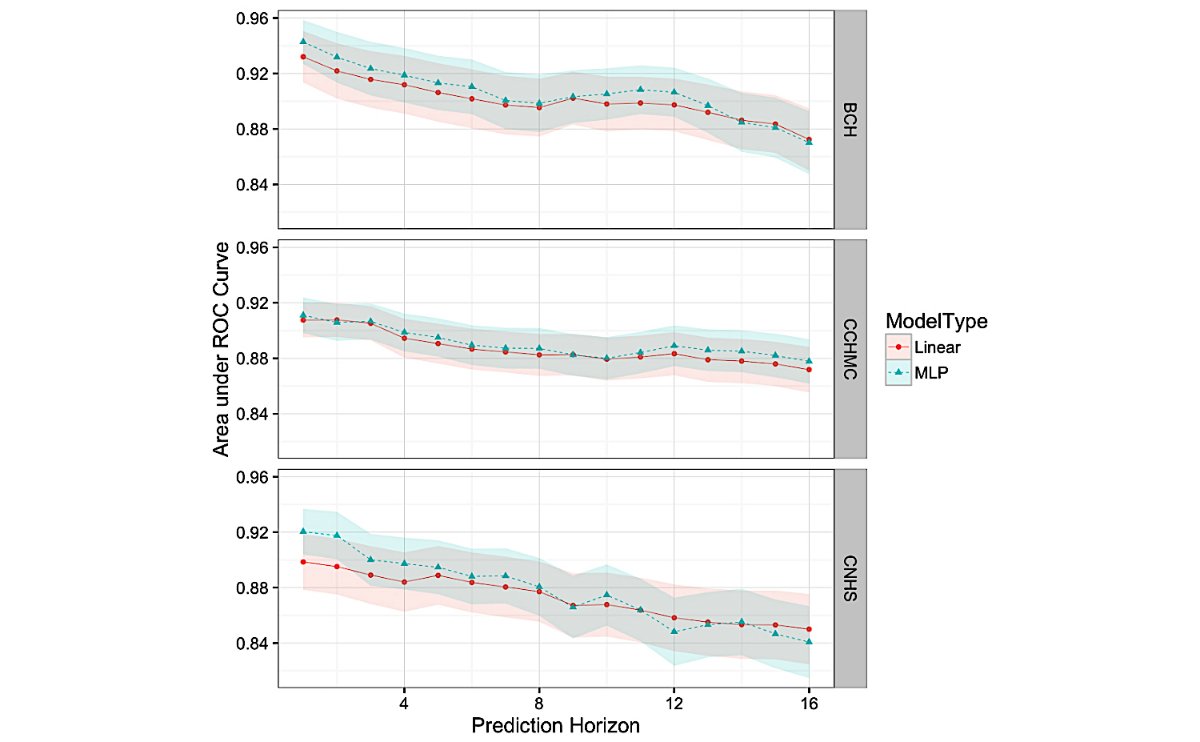
Best regularized logistic regression (linear) model in comparison with a multilayer perceptron (MLP) across different prediction horizons. ROC: receiver operating characteristic; BCH: Boston Children’s Hospital; CCHMC: Cincinnati Children’s Hospital and Medical Center; CNHS: Children’s National Health System.

A final set of experiments compared the performance of MLP and regularized logistic regression models, shown in Figure 5. The MLP models perform slightly better for shorter prediction horizons at BCH and CNHS.

**Table 4:** Evaluation results across all three institutions with various feature sets using a fixed prediction horizon of 6 hours for both training and testing.

Feature set		auROC^a^ (95% CI)	Specificity at 0.8 sensitivity (95% CI)	PPV^b^ % at 0.8 sensitivity (95% CI)
**CCHMC**				
	All features (MLP^c^)	0.890 (0.875-0.904)	0.805 (0.774-0.837)	5.19 (4.48-6.19)
	All features	0.886 (0.871-0.901)	0.811 (0.773-0.839)	5.45 (4.47-6.30)
	All-Vitals	0.881 (0.866-0.896)	0.784 (0.749-0.830)	4.67 (4.04-5.95)
	All-Labs	0.878 (0.862-0.893)	0.802 (0.762-0.830)	5.17 (4.26-5.64)
	All-Acuity	0.880 (0.865-0.895)	0.791 (0.761-0.820)	4.91 (4.23-5.63)
	All-Assessments^d^	0.865 (0.849-0.880)	0.763 (0.718-0.791)	4.29 (3.59-4.85)
	Vitals^d^	0.751 (0.728-0.775)	0.539 (0.470-0.599)	2.21 (1.91-2.53)
	Labs^d^	0.651 (0.618-0.685)	0.315 (0.263-0.403)	1.47 (1.37-1.70)
	Acuity^d^	0.746 (0.719-0.774)	0.551 (0.474-0.618)	2.24 (1.93-2.65)
	Assessments^d^	0.846 (0.828-0.865)	0.738 (0.691-0.775)	3.88 (3.28-4.50)
**BCH**				
	All features (MLP)	0.911 (0.891-0.930)	0.875 (0.834-0.908)	7.73 (5.97-10.3)
	All features	0.902 (0.880-0.923)	0.873 (0.832-0.898)	7.66 (5.90-9.36)
	All-Vitals	0.902 (0.882-0.922)	0.863 (0.807-0.901)	7.14 (5.18-9.62)
	All-Labs	0.880 (0.857-0.903)	0.813 (0.722-0.874)	5.33 (3.65-7.71)
	All-Acuity	0.884 (0.862-0.907)	0.831 (0.773-0.878)	5.87 (4.44-7.95)
	All-Assessments	0.885 (0.862-0.909)	0.855 (0.798-0.889)	6.77 (4.96-8.67)
	Vitals^d^	0.732 (0.699-0.765)	0.479 (0.409-0.579)	1.98 (1.75-2.44)
	Labs^d^	0.803 (0.771-0.835)	0.601 (0.518-0.665)	2.57 (2.14-3.05)
	Acuity^d^	0.812 (0.782-0.842)	0.590 (0.515-0.722)	2.51 (2.13-3.65)
	Assessments^d^	0.814 (0.788-0.842)	0.668 (0.543-0.738)	3.08 (2.25-3.87)
**CNHS**				
	All features (MLP)	0.890 (0.872-0.910)	0.771 (0.718-0.826)	4.40 (3.62-5.71)
	All features	0.884 (0.862-0.905)	0.803 (0.740-0.863)	5.08 (3.89-7.14)
	All-Vitals	0.899 (0.879-0.919)	0.856 (0.805-0.887)	6.82 (5.13-8.53)
	All-Labs	0.869 (0.845-0.893)	0.761 (0.676-0.840)	4.22 (3.15-6.18)
	All-Acuity	0.866 (0.842-0.890)	0.761 (0.678-0.823)	4.22 (3.17-5.62)
	All-Assessments^d^	0.853 (0.828-0.879)	0.700 (0.635-0.788)	3.39 (2.81-4.73)
	Vitals^d^	0.722 (0.689-0.755)	0.471 (0.412-0.569)	1.95 (1.76-2.39)
	Labs^d^	0.700 (0.661-0.740)	0.458 (0.359-0.533)	1.91 (1.62-2.21)
	Acuity^d^	0.735 (0.695-0.775)	0.345 (0.276-0.451)	1.58 (1.43-1.88)
	Assessments^d^	0.844 (0.818-0.871)	0.683 (0.629-0.745)	3.22 (2.76-3.97)

^a^Area under the receiver operator characteristic curve.

^b^Positive predictive value.

^c^MLP: multilayer perceptrons.

^d^Indicates results that are statistically significant compared to the best result for each institution (DeLong test, *P*<.05).

## Discussion

### Analysis of Results

Across all three institutions, our best models generally use all available features. The results show a somewhat consistent pattern across institutions, with the CNHS results generally lower, possibly beacause of less available data. The MLP provides a nonstatistically significant, but consistent, benefit over the linear model in terms of area under the ROC curve. Noteworthy is how the combination of the four different types of features generally provides the best performance, though we do note that removing vitals from the feature sets does not affect the BCH model and, in fact, slightly improves the CNHS model. Nursing assessments provide a strong indication of future deterioration, a finding that holds across all three institutions. This finding is consistent with recent work predicting sepsis that demonstrated significant benefits to utilizing text comment fields [24]. We anticipate that with additional labeled data, the nonlinear MLP model may outperform the logistic regression model. Recent work at predicting deterioration has demonstrated the utility of nonlinear models for predicting deterioration [14], when sufficient data are available. These results are encouraging, showing that a complex MLP with three hidden layers can be regularized sufficiently to avoid overfitting.

Features based on laboratory results, acuity scores, and nursing assessments differed across the three hospitals. These differences were because of the fact that some types of clinical data, nursing assessments in particular, lack a one-to-one mapping across institutions. In addition, vocabularies differ across EHR systems and institutions. For example, one institution might have a nursing assessment “Level of Consciousness” while another abbreviates it to “LOC.” In a similar vein, the values (eg, “drowsy,” “sleepy,” and “alert”) are institution-specific terms, some of which may not map to values at another institution. Rather than attempting to normalize all these clinical elements to the same vocabulary, features were constructed by simply taking the attribute-value pairs as they were realized in the EHR, directly from the corresponding database fields. This has an advantage of reducing the time and labor involved for building a model for new institutions’ EHR systems as it obviates the need to map to a standard feature vocabulary. On the downside, however, each model is specific to a single institution.

When considering deploying deterioration prediction models in the hospital setting, a natural question arises as to the robustness of models across different prediction horizons. For example, if the model is trained to forecast deterioration 10 hours in advance but a patient is, in fact, just 2 hours away from a deterioration event, how well might the model perform? Not surprisingly, our results here demonstrated that ideally models should be trained and used to predict deterioration at a fixed horizon. For example, models trained at predicting deterioration only a few hours away perform very poorly at predicting deterioration 10 to 16 hours prior.

Most previous methods to detect deterioration are more limited than ours. The use of early warning scores, such as the Pediatric Early Warning Scores (PEWS) [25] and Children’s Hospitals Early Warning Score (CHEWS) [26] to assess the severity of a patient’s illness can provide warnings up to 11 hours before code and rapid response team (RRT) events [27]. Yet, these scores require manual entry by nurses and only consider small sets of clinical elements. Other work predicting deterioration uses markedly smaller feature sets than ours [12,14] and make use of 29 predictor variables. The Rothman Index (RI) uses 26 variables [28]. In contrast, we have upwards of 4000 predictor variables across four different types of clinical elements. We believe our rich set of predictor variables not only improves the accuracy of our models but increases their robustness to missing data. Indeed, removing any single feature group only mildly degrades the models’ accuracies, except for the case of nursing assessments. The RI [29-31] and the pediatric RI [32] use stepwise logistic regression for the purpose of predicting 1-year postdischarge risk of mortality and other adverse outcomes. It demonstrated the usefulness of including nursing assessments in predicting patient outcomes; however, it is not used to predict unplanned ICU transfers.

Other previous research also focused on physiologic patient characteristics to predict deterioration. Zhai et al [12] developed an EHR-based logistic regression algorithm to predict escalations to the pediatric ICU (PICU) in the first 24 hours after admission from the emergency department (ED). This work highlights several clinical elements that can be leveraged and while the study focuses on pediatric patients, it limits the patient population to only those who had an unplanned transfer to the ICU within 24 hours. Although direct comparisons are not possible because of different experimental conditions, we note Zhai et al [12] achieved 0.912 area under ROC, predicting deterioration 1 hour in advance. Churpek et al [14] obtained lower results (0.79 area under ROC); their prediction horizon ranges from 8 to 16 hours, and they make use of fewer clinical elements than our models. Recent work by Horng et al [24] predicted the occurrence of infection for purposes of sepsis clinical decision support, showing the importance of text analysis in conjunction with vitals for the task.

In contrast to previous studies, we looked carefully at a range of prediction horizons. Zhai et al [12], included predictions with a fixed horizon of 1 hour, whereas in the study by Churpek et al [14], the horizon effectively varied from 8 to 16 hours. Understanding the model’s predictive power at specific horizons is necessary to determine how frequently the model should be invoked to provide a new risk assessment for deterioration. Here, our model shows robustness to longer horizons, meaning that it may prove beneficial even in settings in which the model can only be run infrequently because of strains it may place on EHR system infrastructure.

### Limitations

There are many directions for future work. Improved methods for handling the nonstationary properties and sampling bias underlying health care data may provide better features through alternative parameterizations of time such as sequence time [33]. Across time scales of months or years, there is potential for data drift as patient populations and practice within the hospital setting change. Methods to detect data drift [34] and ameliorate them [35,36] would increase robustness and provide indications as to when models need to be retrained. Relatedly, models that capture the nonindependent sequence of predictions over time for the same patient, in a state-space or Markov model, may perform better and indicate trends.

In some cases models performed slightly better at longer prediction horizons; we hypothesize some of these trends are caused by noisy or missing inputs. Better features such as those derived from procedures, medication ordering, and administration may provide measures of the patient’s complexity and acuity. Finally, rich information is present in various free-text fields [37,38,24] that may provide indicators of clinician concern.

Methods for providing explanations of model predictions in terms of the predictor variables present may have benefits in terms of validation and clinician acceptance [15]. On the other hand, minimizing labor-intensive feature extraction altogether is an interesting avenue to explore. Specifically, deep learning techniques [39,40] that help to learn representations automatically appear promising.

Adjustment of the outcome variable itself is another area for refinement. Many patients not identified as cases, as they were never transffered to the ICU, could be considered cases by virtue of their potential to have resulted in a deterioration event, had interventions not occurred. Expanding the cases to include patients based on certain interventions may be worth exploring. Another formulation would be to train the model to predict deterioration for some *interval of time* in the future, for example, 4 to 6 hours. This may improve the robustness of the model. Survival analysis based on hazard models is another approach where the goal is to measure the time until deterioration, yet challenges arise from censoring [41] and competing events [42] based on the fact that many patients never go on to have a deterioration event. Finally, in cases where the outcome variable of interest can be observed (eg, acuity scores) or computed (eg, sequential organ failure assessment [SOFA], scores for sepsis [43,44]) as a scalar value at various points in time from EHR retrospectively, deterioration could be formulated as a *forecasting* problem. Although forecasting models are inherently more complex (as they provide a series of nonindependent predictions), they may provide better interpretability, especially in conjunction with CIs associated with the forecast.

### Practical Implications

Deployed in the hospital setting, this model may supplement existing detection tools in use such as safety huddles or rapid response teams to improve the recognition of patients at risk of experiencing an unplanned ICU transfer. Ultimately, the results of the model could lead clinicians to detect deterioration and act sooner. This may avoid serious events that lead to higher rates of morbidity and mortality. There is also great potential to reduce cost through fewer inpatient days, shorter ICU stays, and fewer and less extreme medical interventions.

### Conclusions

This paper described a machine learning approach to predict deterioration in pediatric patients as indicated by an unplanned ICU transfer by leveraging rich sets of clinical elements in the EHR. Our study, carried out at three separate institutions with different EHR systems, suggests that such approaches to predicting deterioration have a great potential to improve care and reduce costs [5]. By analyzing how prediction quality changes across different prediction horizons, we have provided insight into how such a model would fare in a real clinical setting. In addition, our research suggests that feature-rich, data-driven models may perform at a superior level to existing models reported in the literature based on small numbers of carefully tuned variables. Ultimately, the model output may be integrated in workflows of rapid response teams and safety leads so that deterioration could be recognized earlier.

## Abbreviations

ADT: admission-discharge-transfer
auROC: area under the receiver operator characteristic curve
BCH: Boston Children’s Hospital
CCHMC: Cincinnati Children’s Hospital Medical Center
CDE: critical deterioration event
CHEWS: children’s hospitals early warning score
CNHS: Children’s National Health System
ED: emergency department
EHR: electronic health record
ICD-9: international classification of diseases - 9
ICU: intensive care unit
MLP: multilayered perceptron
NICU: neonative intensive care unit
OR: operating room
PACU: postanesthesia care unit
PEWS: pediatric early warning score
PPV: positive predictive value
RI: Rothman index
RRT: rapid response team
ROC: receiver operating characteristic
SOFA: sequential organ failure assessment
SQL: structured query language
